# The impact of integrated genomic surveillance on non-typhoidal *Salmonella* infection in Australia: an ecological study

**DOI:** 10.1016/j.lanwpc.2025.101592

**Published:** 2025-06-17

**Authors:** Son Nghiem, Nhung Mai, My Tran, Danielle M. Cribb, Liliana Bulfone, Patiyan Andersson, Alireza Zahedi, Tuyet Hoang, Tehzeeb Zulfiqar, Angeline Ferdinand, Katie Glass, Martyn D. Kirk, Vitali Sintchenko, Amy V. Jennison, Benjamin P. Howden, Emily Lancsar

**Affiliations:** a National Centre for Epidemiology and Population Health, The Australian National University, Canberra, ACT, Australia; b Microbiological Diagnostic Unit Public Health Laboratory, Department of Microbiology and Immunology at The Peter Doherty Institute for Infection and Immunity, The University of Melbourne, Melbourne, VIC, Australia; c Department of Microbiology and Immunology at The Peter Doherty Institute for Infection and Immunity, The University of Melbourne, Melbourne, VIC, Australia; d Sydney Institute for Infectious Diseases, The University of Sydney, Sydney, NSW, Australia; e Centre for Infectious Diseases and Microbiology-Public Health, Institute of Clinical Pathology and Medical Research, NSW Health Pathology, Sydney, NSW, Australia; f Public and Environmental Health, Pathology Queensland, Queensland Department of Health, Brisbane, Australia; g Centre for Health Policy, School of Population and Global Health, The University of Melbourne, Melbourne, Australia; h Department of Infectious Diseases, Austin Health, Heidelberg, VIC, Australia; i Centre for Health Services Research, The University of Queensland, Brisbane, Australia; j Centre for Pathogen Genomics, The University of Melbourne, Melbourne, VIC, Australia; k Department of Health Economics Wellbeing and Society, The Australian National University, Canberra, ACT, Australia

**Keywords:** Whole genome sequencing, Genomic surveillance, Australia, Salmonella infections, Ecological study

## Abstract

**Background:**

Whole Genome Sequencing (WGS) is a powerful technology for monitoring and detecting outbreaks of infectious pathogens, including non-typhoidal *Salmonella* (NTS). Despite its higher cost than traditional typing methods, WGS offers numerous advantages, including higher resolution and potentially quicker turnaround time. However, evidence regarding its effectiveness in NTS surveillance has predominantly stemmed from micro-simulations or small-scale data. Notably, a recent systematic review identified a lack of real-world, large-scale evidence on the impact of WGS application in NTS surveillance. Our study fills this gap by estimating the effects of WGS on NTS surveillance in Australia using national notifiable disease datasets.

**Methods:**

The main dataset was the National Notifiable Diseases Surveillance System (NNDSS) for NTS from 2009 to 2024. The treatment variable was defined as a binary variable representing the period when WGS was implemented in each jurisdiction of Australia. To minimise the effects of unobserved confounders, we employed a two-stage difference-in-difference (2sDiD) approach. This method estimated the parameters of state and period fixed-effects and then adjusted the observed outcomes from those fixed-effects in the first stage. The average treatment effect was obtained in the second stage by regressing the adjusted outcome against the treatment in the second stage. We also conducted a sensitivity analysis using a multi-period DiD model with a double machine-learning estimator.

**Findings:**

Compared to the pre-WGS periods, the introduction of WGS was associated with an average of 11.6% reduction in NTS cases when a static specification was applied. Results of a dynamic specification were slightly higher, with a 12.7% reduction in NTS cases after WGS. The estimated effects increased to 17.5% when a multi-period DiD model with a double machine learning estimator was applied.

**Interpretation:**

Our study shows that WGS was associated with a significant reduction (11.6%–17.5%) of NTS cases in Australia. Using the cost and break-even point of NTS from previous Australian studies, our findings suggest that WGS is associated with 7200–10,900 cases of NTS averted, saving US$11.3 m–US$17.0 m per year.

**Funding:**

Australian National Health and Medical Research Council, Medical Research Futures Fund (FSPGN00049), and Investigator Grant (GNT1196103) to BPH.

**Research in context**

**Evidence before this study**

We recently conducted a systematic review^1^ of economic evaluation studies on the application of whole genome sequencing (WGS) in pathogen surveillance. We found that only 19 studies examined the effects of WGS on pathogen surveillance. Among these, only two studies estimated the effects of WGS on the surveillance of non-typhoidal *Salmonella* (NTS).^2^^,^^3^ One study analysed annual surveillance data in the USA^2^ and found that WGS was associated with a reduction of 1.5% NTS illnesses per year and a saving of $US97 million. However, this study could not consider potential seasonal effects and unobserved confounders between states. A Canadian outbreak study^3^ estimated that WGS was associated with a reduction in NTS-related illnesses of 50%–70% and a saving of $US54 m–$US75 m if the turnaround time that WGS contributes to early public health responses was 2 months and 3 months, respectively.

**Added value of this study**

This study analysed a large-scale longitudinal surveillance data set using the latest analytical methods in difference-in-difference and machine learning to minimise the potential biases from unobserved confounders. Our findings show that the application of WGS in Australia was associated with a reduction of NTS-related illnesses of 11.6%–17.5%.

**Implications of all the available evidence**

WGS is an emerging technology that has been adopted in pathogen surveillance; however, costs are potentially limiting its broader application. Despite the cost of WGS being higher than conventional typing methods,^4^ our findings of 11.6%–17.5% reduction were substantially higher than the break-even point of 4.2%, resulting in at least 7200–10,900 cases of NTS infection averted or US$11.3–US$17.0 million saved annually.

## Introduction

Foodborne diseases present a substantial global economic burden, resulting in total annual infections of more than 600 million and more than 400,000 deaths.^5^ In Australia, foodborne diseases account for 4.7 million illnesses, costing $1.7 billion per year^6^ (US dollars 2024 prices are used in this study, and monetary units across studies were converted using the price index and exchange rate^7^). Among foodborne pathogens, non-typhoidal *Salmonella* (NTS) is one of the most common and costly pathogens in human health, with 62,000 cases costing an estimated $97 million per year in Australia.^6^

Whole Genome Sequencing (WGS) is a transformative technology across various fields, including pathogen surveillance and outbreak investigation.^8^ On the one hand, WGS offers many advantages over traditional typing methods for characterising pathogens, including higher resolution, greater precision, quicker turnaround time, and richer analytical results that potentially inform more targeted public health responses. On the other hand, at the laboratory level, WGS often incurs higher costs than traditional typing methods.^4^

Despite its widespread application, empirical evidence on the effects of WGS-enabled disease surveillance remains scarce. This evidence is critical to ensure sustainable funding and optimised implementation. As revealed by recent systematic literature reviews,^1^^,^^9^ existing evidence is limited to small-scale investigations or micro-simulations. One exception was a study by Brown et al.,^2^ which evaluated the impacts of WGS on three enteric pathogens (*Escherichia coli*, *Listeria*, and NTS) using US surveillance data from 1999 to 2019. They found that WGS was associated with a reduction of 1.5% in NTS cases per year. However, this study analysed annual national aggregated data and, therefore, could not account for potential seasonal and state fixed-effects on pathogen infections.

WGS has been used in pathogen genomics in Australia for almost 10 years. The first initiative was the establishment of the New South Wales Pathogen Genomics Partnership (NSW-PGP) in 2015. The COVID-19 pandemic accelerated the expansion of WGS with the development of AusTrakka as Australia's national genomics surveillance platform.^10^ However, the progress of WGS implementation varied considerably by jurisdictions. For example, the Australian Capital Territory has not introduced WGS, while Tasmania and the Northern Territory only used WGS in 2022 and 2023, respectively. Differences in resources and workforce capacities contributed to the patchy implementation of WGS in pathogen surveillance across jurisdictions in Australia.^11^ Also, traditional typing methods are implemented alongside WGS, mostly for cost efficiency. The complexity of WGS's gradual implementation and its usage alongside traditional typing methods makes the estimation of its impact challenging.

This study aimed to examine the effects of WGS using national surveillance data in Australia. We also applied a novel statistical model to minimise the effects of unobserved confounders resulting from states or time period heterogeneity. This study is part of a comprehensive national program investigating pathogen genomics for public health in Australia—the Australian Pathogen Genomics Program (AusPathoGen)^12^- a world-first translational research model for integrating pathogen genomics technology into infectious disease response. AusPathoGen is focused on the surveillance of 14 pathogens with a high burden of disease in Australia. Importantly, NTS was identified as a major national priority for genomics implementation in the AusPathoGen project.

## Methods

### Data sources

Non-Typhoidal Salmonella infections are one of 76 nationally notifiable conditions in Australia. Doctors and laboratories are required under State and Territory public health legislation to report cases to one of eight jurisdictional health departments, depending on where the case was diagnosed. The data are aggregated in the National Notifiable Diseases Surveillance System (NNDSS). In this study, we analysed the weekly NNDSS data on notified NTS cases in Australia from January 2009 to January 2024. The NTS surveillance data for the Australian Capital Territory (ACT) were not publicly available in the NNDSS. Hence, we collected this data set from the ACT Department of Health using a request for data releases under the Chief Executive Decision CED09-004. We augmented disease notification data with socioeconomic data collected from the Australian Bureau of Statistics, including the index of relative socioeconomic disadvantage (IRSD), the index of education and occupation (IEO), and the index of economic resources (IER) to control for their effects on NTS infections. These indices are proxied for potentially unobserved factors such as food consumption behaviour and social interactions, which may, in turn, affect the risk of NTS infection.

Our treatment variable is defined based on the time that WGS-based genomic surveillance for NTS was implemented in each jurisdiction of Australia. Although alternative typing methods are still in use by public health laboratories (PHLs) across states and territories, we expect that WGS could still create significant effects on outcomes of interest (e.g., rate of pathogen infections) due to the richer and higher precision information provided. The period when WGS was implemented for pathogen surveillance, including NTS, was collected through surveys distributed to all participating PHLs in Australia.

#### Ethical approval

The research ethics of this study were approved (No. 2022/407) by the Human Research Ethics Committee at the Australian National University on 22 November 2022.

#### Role of the funding source

Australian National Health and Medical Research Council, Medical Research Futures Fund (FSPGN00049), and Investigator Grant (GNT1196103) to BPH. The funding was provided to support activities of the AusPathogen project, including this study.

### Statistical analysis

The impacts of WGS implementation may be affected by unobserved confounders across states and over time. For example, during the WGS implementation periods, Australia also introduced public health policies that could affect NTS infection, such as the foodborne illness reduction strategy 2018–2021,^13^ the biosecurity control order,^14^ and the trial of Salmonella *Typhimurium* vaccine in laying hens.^15^ To address this issue, we applied a two-stage difference-in-difference (2sDiD) method.^16^ In the first stage, parameters of state and period fixed-effects were estimated using never-treated (i.e., have not implemented WGS yet) and not-yet-treated (i.e., periods before WGS) observations. The outcome (i.e., logarithm of NTS cases/1 M population) was then adjusted using the estimated state and period fixed-effects parameters. The adjusted outcome, which eliminates the potential effects of unobserved confounders fixed within a state or an observation period (i.e., weeks), contains only treatment effects and possible measurement errors, which are represented by the error terms. Thus, we could control for confounders within a state or a week during the study period (e.g., COVID-19, NTS vaccine, and public health responses) by using seven parameters of state fixed effects and almost 800 weekly fixed effects. In the second stage, the effects of WGS on NTS infections were estimated by regressing the adjusted outcome on the treatment variable (i.e., WGS).

In addition to the static specification, where average treatment effects (ATEs) are assumed to be constant for the whole treatment period, we also applied a dynamic specification to allow treatment effects to vary over time. In this specification, treatment effects were estimated for each observed period using the period preceding the treatment (i.e., t = −1) as the reference. The ATEs were then estimated by aggregating across states and each treatment period.

For sensitivity analysis, we applied a multi-period DiD model^17^ with a double machine learning (DML) estimator.^18^ The multi-period DiD model^17^ estimates treatment effects for groups of states that introduced WGS at the same period, then weights the estimated group effects to obtain the ATEs for Australia. We applied a double machine learning model to the multi-DiD estimator to allow for potential interactions and non-linear relationships between covariates.^19^ The causal inference of the DML model^18^ was based on the Frisch-Waugh-Lovell (FWL) theorem^20^ and can be classified into two stages. The first stage applies a machine learning model to obtain the residuals of the outcome against covariates (e.g., state fixed-effects, period fixed-effects, and socioeconomic advantage indices); and the residuals of the treatment against the selected covariates. The second stage applies a machine learning model using these two residuals to estimate the effects of WGS on NTS infection. In this study, we used a random forest machine learning model in both stages. To further test the sensitivity of findings to estimation methods, we also conducted a traditional two-way fixed-effects (TWFE) model, which is a generalised linear regression that controls for state fixed-effects and period fixed-effects.

We also estimated the number of cases averted and the net benefit of WGS using the estimated parameters of WGS effects, the annual number of infections that include under-reporting,^6^ and the total costs of NTS in Australia.^4^^,^^6^ The analysis was conducted in R (Version 4.3)^21^ programming language using *did2s*^22^ and *DoubleML*^23^ packages.

## Results

### The NTS rates before and after WGS implementation

Among eight states and territories of Australia, the earliest WGS adopter was Victoria, which introduced the technology in January 2016, followed by New South Wales (October 2016), Queensland (April 2017), Western Australia (January 2018), South Australia (November 2018), Northern Territory (NT; June 2022), and the latest adopter was Tasmania where WGS was introduced in August 2023. The Australian Capital Territory (ACT) was the only jurisdiction that had not implemented WGS at the time of this study. However, laboratory interviews showed that the ACT still benefited from WGS by sending their samples to other states for sequencing and analysis, which makes it difficult to classify this state as a comparator. Given the ambiguity of the ACT regarding WGS adoption, we exclude this state from data analysis.

While the NNDSS data were presented as the number of NTS infections per week, we selected the rate of infection to adjust for differences in the population size between states (Table 1). After adjusting for population, the trend of NTS infection was a sharp decline from 16 cases per 1 million population to 10 cases per 1 million population. We selected the logarithm of cases per 1 million population as the outcome variable to further minimise the potential effects from outliers in observed data (see Supplementary Figure A1). The additional benefit of the logarithm transformation is that the estimated parameter is directly interpreted as relative changes in NTS infection after WGS adoption. The logarithm of cases per 1 million population shows a declining trend from 2.5 logarithm points before WGS to 2.1 logarithm points after WGS. The reduction was only significant among the top 20 serovars (i.e., *Typhimurium (and its variants)*, *Enteritidis*, *Virchow*, *SaintPaul*, *Weltevreden*, *Paratyphi B var Java*, *Wangata*, *Infantis*, *Hvittingfoss*, *Birkenhead*, *Bovismorbificans*, *Chester*, *Stanley*, *Aberdeen*, *Muenchen*, *Agona*, *Mississippi*, *Waycross*, *Newport*, *Anatum*), which account for 72% of the total NNTS infections in the study period. This observation could be because, theoretically, WGS can be applied to all serovars. Still, in practice, public health laboratories prioritise sequencing serovars, which are the most prevalent and, hence, most likely to cause outbreaks.

**Table 1:** Descriptive statistics of NTS notifications.

Outcomes	Pre-WGS (duration: 279 weeks)	Post-WGS (duration: 169 weeks)	p-value
Cases/1 M population/week	16.44	10.26	<0.001
Logarithm of cases/1 M population/week	2.51	2.14	<0.001
Top 20 common serovars	2.09	1.70	<0.001
The remaining serovars	1.09	1.07	0.474

Note that p-values in Table 1 were generated using a t-test and did not account for any covariates or potential confounders that affect the outcomes or WGS adoption between states.

**Fig. 1:**
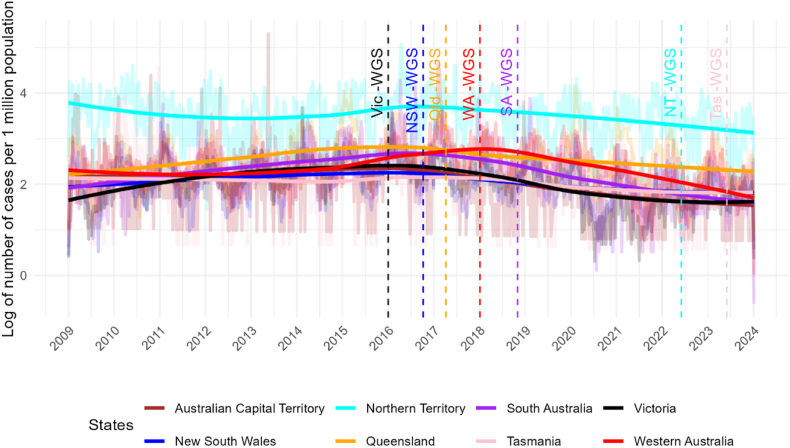
Logarithm of NTS-infected cases/week/1 M population by states and WGS status.

The smoothed time series plot of log NTS cases per 1 million population by states showed a consistent reduction trend across states from 2016 to 2024 when most states introduced WGS (Fig. 1). A t-test shows a significant difference in the logarithm of NTS infection rate by WGS, with the sole exception of the Northern Territory, where WGS was only implemented in 2022. Also, the differences were significant only for the top 20 serovars (see Supplementary Table A1 for details). There are distinct differences in salmonellosis epidemiology between states and territories, with southern parts of the country dominated by predominantly foodborne Salmonella serotypes, such as S. Typhimurium and in the northern parts of the country, higher rates of environmentally mediated Salmonella serotypes. Additionally, there is much greater seasonality in southern states compared to tropical northern parts of the country. Despite the time series plot being a rudimentary visual analysis, this plot suggests that WGS is associated with a reduction in NTS cases, but the effect varies across states and over time. Thus, controlling for state and period fixed-effects in a dynamic specification would result in a more reliable estimate of WGS effects.

### Main findings

Table 2 presents the estimates from the 2sDiD model for both static and dynamic specifications. Overall, we find that WGS introduction was associated with a significant reduction in NTS cases. The finding from the dynamic specification indicates a significant average reduction of 12.7% of NTS cases per 1 million population after the introduction of WGS. However, the reduction was only significant for the top 20 serovars, with an average effect of 28.1%. For the remaining serovars, the finding suggests a potential increase in the NTS rate after WGS by 14.6%, but this estimate is not statistically significant. The finding of the static specification was slightly lower at a 11.6% reduction. A sub-group analysis confirmed that the reduction was only significant for the top 20 common serovars, with an average reduction rate of 26.7%.

**Table 2:** Effects of WGS on NTS infection in Australia.

Models	Parameters	p-value	Changes
*Baseline: Two-stage DiD*			
Static specification	−0.12	0.03	−11.6%
Top 20 serovars	−0.31	<0.001	−26.7%
Remaining serovars	0.14	0.25	15.0%
Dynamic specification	−0.14	<0.001	−12.7%
Top 20 serovars	−0.33	<0.001	−28.1%
Remaining serovars	0.136	0.09	14.6%
*Sensitivity analyses*			
Double machine learning Multi-period DiD	−0.19	0.03	−17.5%
Top 20 serovars	−0.34	<0.001	−28.6%
Remaining serovars	−0.60	0.16	−45.3%
Two-way fixed effects	−0.08	<0.001	−7.9%
Top 20 serovars	−0.14	<0.001	−13.5%
Remaining serovars	−0.08	0.11	−7.9%

**Fig. 2:**
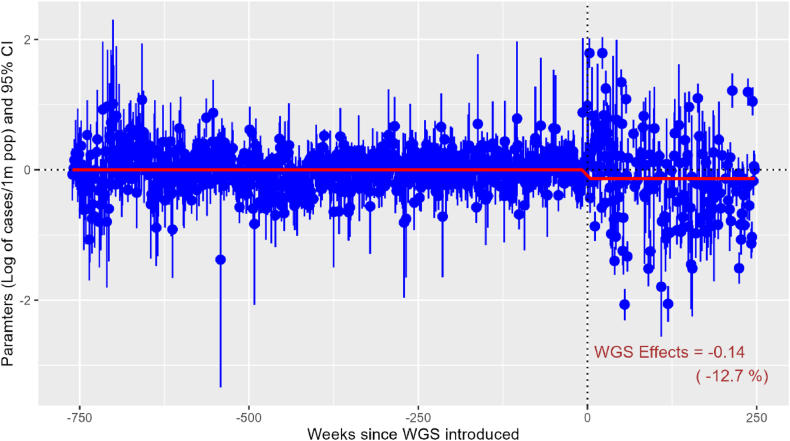
Effects of WGS on NTS cases, two-stage DiD dynamic specification.

One important assumption of the 2sDiD is that there was no pre-trend, meaning that prior to WGS, the number of NTS cases in early and late-treated states evolved similarly after accounting for any observed and unobserved confounders. To test for pre-trend, we conducted an event study, controlling for state fixed-effects and period fixed-effects (Fig. 2). We found no significant pre-trend of NTS cases in the period before WGS. That means, after controlling for state fixed-effects and period fixed-effects, there were no significant differences in NTS cases between early-treated and late-treated states in the pre-WGS periods.

### Sensitivity analysis

To test the robustness of the findings, we applied a DML estimator^18^ in a multi-period DiD model^17^ to examine the effects of WGS on NTS infections. We reached conclusions similar to those of the 2SDiD estimator. However, the DML estimates of ATEs are substantially higher (in absolute value) than those of the 2sDiD estimator, from 11.6% to 17.5% reduction in NTS cases (Table 2). The sub-group analysis also shows that the finding was only significant for the top 20 serovars, with an average reduction of 28.6%. We also estimate a TWFE model, which directly controls for states and period fixed-effects rather than using two stages. While the findings remain similar, the estimated ATEs were only a 7.9% reduction in NTS cases per 1 million population. The sub-group analysis also consistently shows that the reduction was significant only for the top 20 serovars, with an average reduction of 13.5%.

## Discussion

To our knowledge, this study represents the first ecological study that interrogated national-scale surveillance data to estimate the effects of WGS on NTS infections in Australia. This real-world evidence on the positive economic effects of WGS is consistent with findings from recent studies in the USA, Canada and Europe^2^^,^^3^^,^^24^ and will inform policymakers, practitioners and other stakeholders about the benefits of adopting WGS technology in routine foodborne disease surveillance.

Our main findings demonstrate, on average, a substantial 11.6%–17.5% reduction in NTS cases potentially attributable to WGS implementation. Even when a TWFE model was applied, we still found that WGS was associated with a 7.9% reduction in NTS cases. Thus, despite WGS incurring higher costs, the substantially larger effects estimated by our study, compared to the 4.2% break-even point, indicate that WGS can be a cost-saving intervention for NTS surveillance in Australia.^4^ While the NNDSS data show that the number of NTS notifications averaged 12,300 cases per year, it was estimated that this corresponds to 62,000 illnesses per year when under-reporting is taken into account.^6^ Thus, our estimate of the WGS effect (11.6%–17.5%) would translate to 7200–10,900 NTS cases averted per year, when including cases not reported to health authorities. Using the estimated total cost of NTS of $97 million per year,^6^ and the estimated break-even point of 4.2%,^4^ the net benefit of WGS in NTS surveillance in Australia is estimated at $11.3–$17.0 million per year.

Two components of our analysis may have resulted in us underestimating the effect of WGS. First, many Australian jurisdictions continue to use WGS alongside traditional typing methods. Thus, WGS effects could be diluted by those of traditional methods that are still partly applied in practice. Second, the reduced risk of pathogen infection in states with WGS technology also creates positive spillover effects to those without WGS technology through transactions of goods/services or inter-state travel. For example, we are aware that even though the ACT has not yet implemented WGS, the ACT PHL can still access WGS by sending their samples to PHLs in nearby states for sequencing.

Our estimated effects are in the middle range compared to previous studies on the impacts of WGS in NTS surveillance. The Canadian study by Jain et al.^3^ found that WGS was associated with a 50%–70% reduction in the incidence of NTS infection, resulting in a total cost-saving of $54 and $75 million, respectively. When reported cases are used in the estimate, the net benefit of WGS was $4.2 million. Compared to the findings of their study, the magnitude of our finding (11.6%–17.5%) was much more modest. However, their upper-bound estimation (70%) of effects was based on a simulation with the assumption that WGS resulted in detecting an outbreak earlier by three months. This assumption contrasted with the observed evidence from Italy, where WGS was associated with the early detection of an NTS outbreak by two months.^25^ The lower bound estimate of 50% reduction was based on the evidence from a study by Bell et al.,^26^ who reported that WGS reduced the turnaround time for sample analysis by 4–5 days compared to Pulsed-Field Gel Electrophoresis (PFGE) and that quicker turnaround resulted in halving the incidence of illness.

However, our estimated effect is greater than that of Brown et al.^2^ for the USA, who found that WGS was associated with a reduction of 19,800 NTS cases and $97 million in savings of illness burden (or about 1.5% of illness/disease burden) in 2019. The finding of a net saving despite a reduction due to WGS of only 1.5% in this study was consistent with that of Jain et al.,^3^ which found that a modest reduction of 0.6% in cases was enough for WGS to break even in the USA.^24^ Like our study, the authors also control for pathogen fixed-effects, year fixed-effects, and other interventions such as the implementation of the Food Safety Modernisation Act in 2011. However, they could not control for state fixed-effects and potential seasonal effects due to data limitations (i.e., they used aggregated yearly data at the national level).

Our findings are also supported by a case study conducted in Europe and America,^24^ where the authors found that although WGS costs 2–4 times more than conventional methods, the percentage of NTS cases avoided to achieve break-even is minimal, ranging from 0.2% (Argentina) to 1.1% (Canada).

Overall, the estimated effects of WGS on NTS infection vary considerably across studies, ranging from 1.5% in the USA^2^ to 70% in Canada.^3^ Various factors may contribute to the substantial differences, including data and methods. For example, Alleweldt et al.^24^ used laboratory-level data, while Brown et al.^2^ used annual national-level data. Due to data differences, the selected methods of analysis are also different. For example, Brown et al. were unable to control for state-fixed effects and weekly fixed effects, as we did, because they used yearly national data. Thus, studies using comparable data sets (e.g., national surveillance) and similar analysis methods are required to make meaningful comparisons and potentially synthesise findings across countries.

The main limitation of our study is due to its ecological design. Observational data may be contaminated by unobserved confounders. For example, certain policies (e.g., NTS vaccination of poultry, public health policies, and public health responses to the COVID-19 pandemic) could also affect the prevalence of NTS in Australia. To minimise the effects of unobserved confounders, we applied the latest methodological treatments (i.e., 2sDiD, DML multi-period DiD) to minimise the effects of time-invariant unobserved confounders in the whole study periods (i.e., 800 weeks from January 2009 to January 2024), and unobserved confounders that are fixed within each of the main eight states and territories in Australia. The DML estimates also allow us to control for potential interactions or non-linear relationships between state and period fixed-effects. Despite these efforts, our estimates are not able to differentiate the effects of any confounders (e.g., NTS vaccine, public health policies) that happened during the same period as WGS.

### Conclusions

This study estimates the benefits of WGS effects on NTS surveillance using real-world, large-scale data from Australia. We applied the latest development in estimation causal inference methodology to control for potential unobserved confounders across jurisdictions and over time. Our results indicate a substantial 11.6%–17.5% reduction in NTS infections following WGS implementation. This effect translated to 7200–10,900 cases averted or a saving of $11.3–$17.0 million per year. Our findings highlight the significant national benefits of the implementation of sustainable WGS-enabled surveillance for NTS, which can reduce foodborne disease burden and save healthcare costs.

## Contributors

**SN**: conceptualization, methodology, data curation, investigation, formal analysis and writing (original draft and reviewing), verified the data, had access to raw data, final responsibility for the decision to submit.

**DC, NM, MT and LB**: data curation, verified the data, had access to raw data, methodology, investigation, and writing (review & editing).

**PA and AZ**: data curation, investigation, and writing (review & editing).

**TZ and AF**: methodology, investigation, data curation, and writing (review & editing).

**KG, MDK, VS, AVJ, BPH, EL**: Funding acquisition, supervision, methodology, and writing (review & editing).

## Data sharing statement

The SA2 level data used in this paper requires approval from the Department of Health in Australian states and territories. However, state-level data are publicly available from the NNDSS website.

## Declaration of interests

Authors declare that they have no competing interests.

## Figures and Tables

**Supplementary Figure A1, a:**
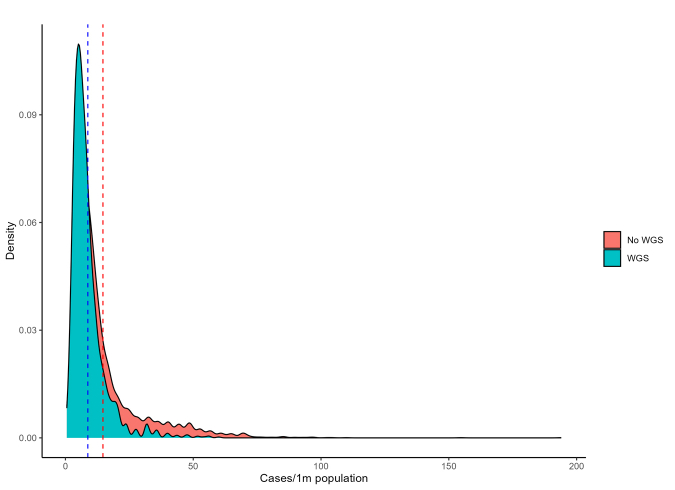
Distribution of cases/1 m population by WGS.

**Supplementary Figure A1, b:**
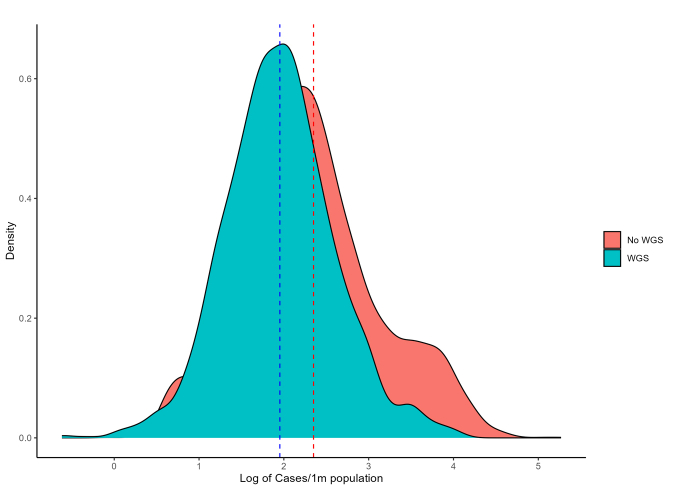
Distribution of logarithm of cases/1 m population by WGS.

## References

[1] Tran M., Smurthwaite K.S., Nghiem S. (2023). Economic evaluations of whole-genome sequencing for pathogen identification in public health surveillance and health-care-associated infections: a systematic review. Lancet Microbe.

[2] Brown B., Allard M., Bazaco M.C., Blankenship J., Minor T. (2021). An economic evaluation of the whole genome sequencing source tracking program in the US. PLoS One.

[3] Jain S., Mukhopadhyay K., Thomassin P.J. (2019). An economic analysis of salmonella detection in fresh produce, poultry, and eggs using whole genome sequencing technology in Canada. Food Res Int.

[4] Ford L., Glass K., Williamson D.A. (2021). Cost of whole genome sequencing for non-typhoidal Salmonella enterica. PLoS One.

[5] World Health Organization (2015).

[6] Glass K., McLure A., Bourke S. (2023). The cost of foodborne illness and its sequelae in Australia circa 2019. Foodborne Pathog Dis.

[7] Shemilt I., James T., Marcello M. (2010). A web-based tool for adjusting costs to a specific target currency and price year. Evid Pol.

[8] Jackson B.R., Tarr C., Strain E. (2016). Implementation of nationwide real-time whole-genome sequencing to enhance listeriosis outbreak detection and investigation. Rev Infect Dis.

[9] Price V., Ngwira L.G., Lewis J.M. (2023). A systematic review of economic evaluations of whole-genome sequencing for the surveillance of bacterial pathogens. Microb Genom.

[10] Hoang T., da Silva A.G., Jennison A.V., Williamson D.A., Howden B.P., Seemann T. (2022). AusTrakka: fast-tracking nationalized genomics surveillance in response to the COVID-19 pandemic. Nat Commun.

[11] Williamson D.A., Kirk M.D., Sintchenko V., Howden B.P. (2019). The importance of public health genomics for ensuring health security for Australia. Med J Aust.

[12] Webb J.R., Andersson P., Sim E. (2024). Implementing a national program of pathogen genomics for public health: the Australian Pathogen Genomics Program (AusPathoGen). Lancet Microbe.

[13] Secretariat F.R. (2018).

[14] Chousalkar K., Gast R., Martelli F., Pande V. (2018). Review of egg-related salmonellosis and reduction strategies in United States, Australia, United Kingdom and New Zealand. Crit Rev Microbiol.

[15] Groves P., Sharpe S., Muir W., Pavic A., Cox J. (2016). Live and inactivated vaccine regimens against caecal Salmonella Typhimurium colonisation in laying hens. Aust Vet J.

[16] Gardner J. (2022). Two-stage differences in differences. arXiv.

[17] Callaway B., Sant'Anna P.H. (2021). Difference-in-differences with multiple time periods. J Econom.

[18] Chernozhukov V., Chetverikov D., Demirer M. (2018). Double/debiased machine learning for treatment and structural parameters. Econom J.

[19] Orfanoudaki A., Chesley E., Cadisch C. (2020). Machine learning provides evidence that stroke risk is not linear: the non-linear Framingham stroke risk score. PLoS One.

[20] Peet E.D., Schultz D., Lovejoy S., Tsui F. (2024). The infant health effects of doulas: leveraging big data and machine learning to inform cost-effective targeting. Health Econ.

[21] R Core Team (2022). Computing RFfS.

[22] Butts K. (2021).

[23] Bach P., Chernozhukov V., Kurz M.S., Spindler M. (2024). DoubleML-an object-oriented implementation of double machine learning in R. J Stat Software.

[24] Alleweldt F., Kara Ş., Best K. (2021). Economic evaluation of whole genome sequencing for pathogen identification and surveillance–results of case studies in Europe and the Americas 2016 to 2019. Euro Surveill.

[25] Morganti M., Bolzoni L., Scaltriti E. (2018). Rise and fall of outbreak-specific clone inside endemic pulsotype of Salmonella 4,[5], 12: i:-; insights from high-resolution molecular surveillance in Emilia-Romagna, Italy, 2012 to 2015. Euro Surveill.

[26] Bell R.L., Jarvis K.G., Ottesen A.R., McFarland M.A., Brown E.W. (2016). Recent and emerging innovations in Salmonella detection: a food and environmental perspective. Microb Biotechnol.

