# Supplementary material for: The impact of integrated genomic surveillance on non-typhoidal Salmonella infection in Australia: an ecological study
Source: Lancet Reg Health West Pac. 2025 Jun 17;59:101592. doi: 10.1016/j.lanwpc.2025.101592 (PMC12212105; doi:10.1016/j.lanwpc.2025.101592)
Supplement: Supplementary Table [file mmc1.docx]

**Table A1.** The logarithm of NTS cases per 1M population by states, WGS and serovars

|  | All serovars | | | Top 20 serovars | | | Remaining serovars | | |
| --- | --- | --- | --- | --- | --- | --- | --- | --- | --- |
| States | No-WGS | WGS | p-val. | No-WGS | WGS | p-val. | No-WGS | WGS | p-val. |
| Victoria | 2.14 | 1.91 | <0.001 | 1.87 | 1.56 | <0.001 | 0.74 | 0.76 | 0.57 |
| New South Wales | 2.13 | 1.95 | <0.001 | 1.79 | 1.53 | <0.001 | 0.92 | 0.92 | 0.96 |
| Queensland | 2.59 | 2.47 | <0.001 | 2.22 | 2.03 | <0.001 | 1.40 | 1.47 | 0.12 |
| Western Australia | 2.38 | 2.26 | <0.001 | 1.99 | 1.84 | <0.001 | 1.24 | 1.15 | 0.05 |
| South Australia | 2.40 | 1.91 | <0.001 | 2.11 | 1.48 | <0.001 | 0.91 | 0.76 | 0.01 |
| Northern Territory | 3.53 | 3.20 | <0.001 | 2.76 | 2.09 | <0.001 | 2.68 | 2.50 | 0.09 |
| Tasmania | 2.03 | 1.89 | 0.34 | 1.70 | 1.52 | 0.24 | -0.31 | 0.31 | 0.07 |
